# Supplementary material for: Are carbon emissions trading and green financial instruments synergistic? -Comprehensive quantitative research based on content analysis
Source: PLoS One. 2024 Mar 7;19(3):e0298601. doi: 10.1371/journal.pone.0298601 (PMC10919601; doi:10.1371/journal.pone.0298601)
Supplement: S1 Data — (ZIP) [file pone.0298601.s001.zip › Data/地级市Z_F工作报告.zip!/┴╔─■/░░╔╜╩╨/└┤╫╘╣1⁄2╓┌║┼í╛┬φ┐╦╩2╛▌═°í┐_2007.docx]

各位代表：

现在，我代表市人民政府，向大会作政府工作报告，请予审议，并请市政协各位委员提出意见。

一、2006年政府工作回顾

2006年是我市在“十五”的坚实基础上经济社会发展取得新突破的一年。我们在市委的领导下，在市人大和市政协的监督支持下，认真贯彻市第十次党代会精神，紧紧围绕加速经济隆起、科学发展鞍山主题，按照“三高两低一稳定”的总体要求，坚持一手抓发展、一手抓民生，一手抓空间、一手抓项目，较好地完成了市第十三届人大第四次会议确定的各项任务，在“十一五”起步之年实现了良好开局。

预计，全年实现地区生产总值1160亿元，按经济普查后“新口径”比上年增长16.5%；万元GDP能耗下降5.2%；地方财政一般预算收入完成56.1亿元，扣除不可比因素增长11.7%；完成全社会固定资产投资407亿元，增长30%；实现规模以上工业增加值420亿元，增长18.1%；实际利用外商直接投资1.3亿美元，增长87.4%；引进内资268.3亿元，增长2.5倍；外贸出口总额突破20亿美元，增长51.7%；实现社会消费品零售总额253.5亿元，增长14.5%；城市居民人均可支配收入达到10760元，农民人均纯收入达到5280元，分别增长13.7%和11.2%。

回顾过去一年，我们主要做了以下七个方面工作：

（一）加快园区和项目建设，经济隆起的新格局全面拉开

坚持一手抓空间、一手抓项目，园区建设取得突破性进展，基础设施全面形成，大批项目开工建设。

发展空间格局初步形成。制定了《城市空间发展布局规划》、《腾达工业走廊规划》等九项专题规划，按照工业进园区、商业进城区、民居进小区的方向，不断调整城市功能布局。规划产业发展空间56平方公里，其中工业发展空间43平方公里，拓展有效工业空间15平方公里。全市已经形成东部高新区、西部达道湾工业区、南部汤岗子农高区、北部灵山工业区、中心城区现代商贸生活区等东西南北中五大发展空间。园区建设全面推进，开工建设了腾达大道、建材街等26条63公里长的道路，引进辽宁电力和国电集团等企业投资8.3亿元建设供电和供热设施。工业园区已经具备项目入驻的良好条件，进区项目197个，总投资185亿元。

项目建设取得良好成效。全市规模以上项目总数达到1005个，总投资1500亿元。其中，开工建设和竣工投产679个。谋划招商英国艾立泰克公司150万台汽车发动机、鞍钢60万吨煤焦油、烨联公司不锈钢、瑞士钢铁公司特种钢等180个项目。推进实施鞍钢26万吨有取向硅钢片、20万吨无缝钢管、200-300万吨板材剪切配送中心和意大利依米法比公司与海城艾海公司合资生产滑石深加工、德国雷法公司与海城牌楼镁矿合资生产高纯镁、腾龙公司热电厂等146个项目。开工建设中冶东建公司40万吨钢管、鞍钢重机公司大型船用曲轴、华冶公司大功率变频变压器、宝得钢铁公司二期、惠丰公司精细化工、东亚公司5万吨精密冷轧不锈钢板、翔鹭化纤公司工业丝和帘子布、中钢集团8万吨针状焦、衡业集团改装汽车、香港新世界百货、万科城市花园等460个项目。竣工投产鞍钢西部500万吨板材精品基地、鞍千矿业公司年产1000万吨铁矿石和300万吨精矿粉、中油天宝集团30万吨高频直缝焊管、翔鹭化纤公司民用丝、鞍轮集团全钢丝子午胎一期、千山滑雪场一期、玉佛苑二期等219个项目。高新区当年新开工项目74个，新增规模以上企业50家，地区生产总值和财政收入等主要经济指标均增长35%以上。全市规模以上工业企业新增260家，总数突破1000家，达到1065家。

地企融合促进企业发展。不断深化“地企融合、共兴共荣”发展理念，形成了政府大力支持企业发展，企业积极回报社会的良好局面。完成大营盘村移村再建，及时为鞍钢新上项目提供1.67平方公里空间。在灵山工业区建设中冶东建公司、鞍钢重机公司和鞍钢附企工业园。支持鞍钢、中冶东建公司进行主辅分离。全面接收了鞍钢分离的医疗机构。地方与鞍钢达成共识，共同推进鞍钢附企改革与发展。鞍钢钢铁主业完成重组，实现整体上市，主要产品产量均创历史新高，预计全年铁、钢产量双超1500万吨、钢材产量超1400万吨，实现增加值275亿元，增长19.1%，为鞍山经济社会发展做出了巨大贡献。及时帮助民营企业解决土地、融资等问题，促进了民营经济快速发展。全年民营经济实现增加值592亿元，比上年增长17.4%；实缴税金39亿元，增长21%，占全市税收比重35%，比上年提高10个百分点。

（二）大力发展县域经济，新农村建设迈出较大步伐

县域生产总值实现485亿元，增长17.1%，占全市地区生产总值的41.8%。县域财政一般预算收入完成14.7亿元，增长24.2%。粮食总产量达到141.6万吨，创历史新高。

县域工业不断壮大。市政府安排2000万元，支持海城、台安和岫岩工业园区建设。一市两县新建、扩建县域工业园区12个。海城市形成了“一带、两区、八园”的发展格局，台安工业园和岫岩偏岭菱镁工业园、兴隆工业区不断扩建和壮大。县域工业开工项目超过300个，比上年增加1倍。

农业产业化稳步推进。市政府补贴2000万元，在鞍羊路两侧建设占地2万亩6000多栋大棚的设施农业示范带。市政府出资550万元，在鞍羊路沿线及台安县各村建设220栋公益大棚。海岫路沿线特色农业示范带全面开工建设。完成造林12.45万亩，植树4281万株。全市综合农业机械化水平达50%。

县域服务业加快发展。实施“万村千乡”、“双百工程”、“信福工程”，新增乡村连锁超市325家。积极发展农事旅游，西柳镇、岫岩镇被评为辽宁省特色旅游乡镇。

惠农政策进一步落实。落实农村义务教育“两免一补”政策，减轻农民负担4811万元；发放种粮、良种和农机购置补贴9372万元；市财政用于农村合作医疗支出2166万元。全年农民人均政策性收益96元，比上年增加35元。

农村基础设施建设力度加大，社会事业不断进步。投入8028万元，新建农村公路188.8公里。完成农村饮水工程77处。新增农村自来水受益人口5万人。新增新能源用户3.1万户。建成了市动物疫病控制中心。市财政出资建设了市农产品检测中心、3 个县级检测站、30个生产基地检测点、79个特色农业技术推广站。加强了农村兽医队伍建设。强化了市场检疫和屠宰检疫。

（三）积极解决人民群众最急盼解决的民生问题，努力让发展的成果惠及人民群众

坚持一手抓发展、一手抓民生，着力解决人民群众安居乐业和生活保障问题，为全市人民办的16件实事全面完成。

实现群众安居有新突破。政府主导棚户区和危房改造，保证了房屋质量和按时回迁。上年动迁的27片32万平方米8200户棚户区居民全部回迁。剩余7片25万平方米7400户棚户区居民全部动迁。胜利路沿线等地区28片76万平方米危房全部拆迁，回迁房全面开工建设。

实现群众乐业有新突破。鞍山市被确定为全国就业工作规范化管理试点城市。全年实现实名制就业15.9万人，城镇登记失业率控制在4.98％。动态实现12164户“零就业家庭”至少1人就业，其中1959户实现“双就业”。推进低保对象就业，全年减少低保人员7.8万人，实现了动态管理下的应保尽保。

完善社会保障体系有新突破。全年新增养老保险4.14万人、医疗保险3.94万人、失业保险3.03万人。收缴养老保险金9.2亿元、医疗保险金6.2亿元、失业保险金2.1亿元，分别比上年增长13.6%、20.3%和12.6%。在全国率先创建了城市合作医疗保险制度，重点解决了不具备参加基本医疗保险的国有和集体企业职工医疗保障问题。在全省率先普及了新型农村合作医疗。在全国率先制定实施了《城乡特困群众社会救助暂行办法》，救助困难群众超过12万人次。全面提高了最低工资、最低生活保障金和企业退休人员养老金标准。

解决群众日常生活难题有新突破。政府和企业投入4.5亿元，更新改造锅炉70台，改造供暖管网50公里，较好地解决了供暖温度偏低和南出口23栋“冰棍楼”无供暖设施等供暖问题。投资4250万元，改造部分燃气管网和2950处漏损的进户管线。鞍钢投资改造输往市区的煤气管道，提高了煤气输送能力。协调辽河油田增加了天然气供应量。改善首山水源水质，扩大汤河水源供水范围，解决了部分地区居民饮水安全问题。

解决重大信访问题有新突破。把解决群众的实际问题，作为做好信访工作的出发点和落脚点。采取领导包案、专题会议研究和现场办公等方式解决重大疑难信访案件和群众关心、媒体关注的热点难点问题。全年共解决重大和疑难信访案件632件，积极维护了社会稳定。

（四）深化国有企业改革，正在走出一条解决历史遗留问题的新路

坚持四条标准，规范国企改制。按照产权明晰、各证到位，保险接续、内欠偿还，转换身份、充分就业，制度创新、做强做大四条标准，对以产权转让方式改制的201户企业履约情况进行全面监管。其中，97户企业完成验收，履约的46户企业各证已办理到位，对没完全履约的43户企业限期整改，对不具备条件和严重违约的15户企业重新改制。

按照三个途径，保障职工利益。按照改革与保障并举、并轨与就业并举、改制与发展并举三个途径，解决了8万名职工养老保险接续问题，使4.8万名职工重新就业，通过建立城市合作医疗保险制度解决了困难企业职工医疗保障问题。

主攻五个专项，系统解决历史遗留问题。通过推进对改制企业监管、解决破产企业和“壳”企业遗留问题、解决职工医疗保障问题、解决企业债务问题、做大做强改制企业五个专项，使改制企业协议执行情况得到有效监管，破产企业和“壳”企业难题得到有效破解，职工社会保障和就业问题得到有效解决。政府出资3.9亿元，以打包缩水收购长城公司不良债权方式，为167户企业解除债务14.3亿元。

运用两大办法，筹措国企改革成本。运用政府收购企业资产和政府出资解决“壳”企业问题两大办法，解决职工的“三险一金”和内欠问题。由政府收购企业土地和破产企业未变现资产，用变现增值的收益偿还了部分困难企业和破产企业拖欠职工的“三险一金”和内欠7.5亿元，占总额的70%。政府直接出资解决了35户“壳”企业1万多名职工“三险一金”问题。

（五）加强城市建设与管理，城市面貌发生新的变化

城市建设步伐加快。推进了《鞍山市城市土地利用规划修编方案》和《鞍山市城市热电发展总体规划》的编制工作。王石500千伏变电所投入运行，开工建设了鞍山、龙王、达道湾等10个变电所。引入国电集团投资建设两台30万千瓦发电机组的热电厂项目。改造建设了园林路等城市主次干道59公里。建成深营路西线隧道。改造街巷路24条，改造居民小区50个。改造供气管网11.7公里，自来水管网24.3公里。改造了二一九、孟泰、烈士山公园。城区新建绿地56万平方米，栽植树木20万株。设置和更换路灯2400基。

城市管理逐步规范。广泛开展了市容环境综合整治“百日会战”，城市亮化、绿化、美化、净化水平进一步提升。对西鞍山等重要资源实施了严格保护。实施了17个大气治理和6个废水治理项目。取缔建成区10吨以下燃煤供暖锅炉87台。完成250万平方米抑尘工程，恢复矿山生态破坏面积115万平方米。新增自然保护区1370万平方米。建成区全年空气质量有80%天数达到国家二级标准。

（六）切实加强社会建设，推进了经济社会协调发展

科技创新有新提高。5个国家863计划项目通过验收。新增企业技术中心6家、研发中心和中试基地7家、自主知识产权企业45家、省级高新技术企业11家。建立国家级博士后工作站3个、省级博士后科研基地4个。

教育事业有新成果。将华育等义务教育阶段国有民办学校全部改为国有公办学校，全面取消高收费，保证义务教育阶段国有资产全部用于义务教育。新建7所农村九年一贯制寄宿学校。投入6000万元改善中小学校园环境和教学设施。新建、扩建海城南台高中、岫岩二高中。在省内率先实行农民工子女可报考市内高中政策。在一市两县各建1所职业技术学校。鞍山科技大学更名为辽宁科技大学，新增博士点2个、硕士点12个。鞍山师范学院教学规模进一步扩大。

卫生事业有新发展。建成了紧急医疗救援中心、急救站和网络医院三位一体的医疗救援体系。完成了千山结核医院搬迁。完善了市县乡三级医疗卫生服务体系。政府安排1045万元，新建和改造了29所乡镇卫生院和10所边远地区乡村卫生所。市财政出资按国家标准为54所乡镇卫生院配齐医疗设备。

社区建设有新进展。投入1020万元，改扩建了17处“一站四室”，增加了5个社区服务中心和141个健身场所，实现了每个社区至少有一处居民健身场地。

文化、体育等其他社会事业又有新进步。成功举办了“2006中国森林旅游博览会暨第六届中国鞍山·千山国际旅游节”。全年组织10场大型文化广场演出，深入农村和基层演出1000余场。新建450处城乡体育健身场地。在鞍山电视台开办了手语节目。实施了“光明行动”和“助听”、“助行”工程。人口自然增长率控制在0.7‰。“双拥模范城”通过了省和国家的检查验收。全市共输送1750名优秀青年入伍。建立了市县两级重大食品安全事故应急工作体系，开展了以食品加工、食品流通、餐饮消费环节为重点的食品安全系列专项整治行动。消防和安全生产工作得到了进一步强化。“平安钢都”建设取得新进展。在城乡重点开展了侦破大案要案、打黑除恶、打击“两抢一盗”等系列严打专项行动，确保了全市治安大局的持续稳定。

新闻出版、广播电视、妇女儿童、老龄、民族、宗教、外事、侨务、对台、统计、旅游、接待、史志、档案、气象、地震、人防、民兵预备役等方面工作都有新的进步。

（七）推进体制机制创新，政府自身建设进一步强化

民主法制建设不断增强。坚持政府工作及时主动向人大及其常委会报告，积极与人民政协进行民主协商。建立市政府与市人大、市政协反复督办“滚动式”调度机制，采取书面答复和推进落实“两段式”办理方式，172件人大代表建议和438件政协提案全部办结。向人大常委会提报立法议案2件，废止1件，制定和修改政府规章10件。全面启动“五五”普法工作。

制度建设不断完善。坚持“管、办”分离原则，建立了“决策、执行、监督”三线分离和协调机制。成立了政府投资建设项目招标中心，完善了政府采购中心管理体制，加强了土地储备中心、房产交易中心和产权交易中心建设。

服务水平不断提升。对经济社会和民生等重大事项，采取多种方式征求社会各界意见，倾听群众呼声。规范了审批程序，简化了审批环节。对重点建设项目，实行“一条龙”服务。鞍山市被国家评为“中国投资环境百佳城市”。

队伍建设不断强化。严格落实廉政建设责任制，纠正部门和行业不正之风，加大对政府机关和领导干部的行政监察力度，从源头上治理和预防腐败。

各位代表，过去一年，经过不懈努力，全市正呈现出经济隆起、科学发展的良好态势。这些成绩的取得，得益于市第十次党代会的正确指引，得益于“十五”奠定的坚实基础，得益于市委的正确领导和市人大、市政协的依法监督、民主监督和鼎力支持，得益于鞍钢等中省直单位和驻鞍部队、武警官兵的无私奉献，得益于各位代表、各位委员和全市各族人民的共同奋斗。在此，我代表市政府向全市各条战线的广大干部群众，向各位代表、各位委员、各民主党派、工商联、各人民团体和关心支持鞍山发展的老领导、老干部，向所有为鞍山发展做出贡献的各界人士，表示衷心的感谢和崇高的敬意！

在看到成绩的同时，我们也清醒地认识到鞍山发展还存在一些问题，政府工作还存在许多不足。主要是经济增长方式粗放，乱占滥采滥用资源现象仍然存在，节能降耗和污染减排任务相当艰巨；产业结构不合理，产业链条短，产品精深加工水平低，现代服务业特别是文化产业发展滞后；开放程度低，战略性、牵动性的大项目较少，开放型地区经济发展水平不高；社会事业发展相对滞后，就业和社会保障压力仍然较大，城乡发展不平衡，城乡居民收入差距拉大的趋势没有根本改变；投资环境还需改善，机关工作作风和效率有待改进和提高。对这些问题，我们将在今后的工作中努力加以解决。

二、2007年政府工作任务

根据市委十届二次全会暨经济工作会议的部署，新的一年，政府工作总体思路是:以邓小平理论和“三个代表”重要思想为指导，全面贯彻党的十六届五中、六中全会和中央、省经济工作会议精神，按照市第十次党代会确定的“总量倍增、位次前移、追赶沈大、殷实和谐”的奋斗目标，以科学发展观为统领，突出加速经济隆起、构建和谐鞍山主题，一手抓发展、一手抓民生，一手抓城市、一手抓农村，坚持经济工作以项目为核心，调整优化经济结构，加速转变经济增长方式，强化新农村建设，加快社会事业发展，进一步改善人民生活，努力实现经济社会又好又快发展。

主要预期目标是：地区生产总值增长13%；地方财政一般预算收入增长13.3%；实际利用外商直接投资增长20%，到位内资增长20%；全社会固定资产投资增长20%；社会消费品零售总额增长13%；城市居民人均可支配收入和农民人均纯收入分别增长10%以上；万元GDP能耗下降5%，主要污染物排放量比上年平均减少3％。

按照上述总体思路和预期目标，我们重点要做好以下七个方面工作：

（一）以结构调整为主线，努力实现经济增长速度、质量、效益相协调

着力调整经济结构，切实转变经济增长方式，努力实现经济又好又快发展。

在继续加强精品钢材基地建设的同时，大力发展“三大产业”。一是大力发展钢铁产业。继续为鞍钢的发展创造良好条件，以鞍钢为龙头，以中冶东建公司、宝得钢铁公司、中冶德龙公司、鞍钢附企、中油天宝集团等企业为重点，大力发展钢材深加工产业。以推进东亚公司和烨联公司冷轧不锈钢、瑞士钢铁公司特种钢等项目为重点，进一步优化钢铁产业结构。二是大力发展现代装备制造、矿产品深加工、轻纺工业和其它产业。以鞍钢和鞍钢矿业公司、鞍钢重机公司等企业为龙头，充分依靠中冶焦耐、中冶北方、热能院等中省直科研院所的技术力量，用“鞍山设计”、“鞍山创造”和“鞍山制造”拉动装备制造、高新技术等产业快速发展。以西洋集团、后英集团、北海集团、海城艾海公司等企业为重点，大力发展矿产品深加工产业。以海城翔鹭化纤公司等企业为重点，大力发展纺织业。以鞍钢、辽化为依托，以中钢集团等企业为重点，大力发展石油化工、煤化工产业。

在继续做大做强工业的同时，大力发展现代服务业。一是大力发展商贸物流业。重点推进四达物流园区、鞍钢板材剪切配送中心等生产型服务业。引进家乐福、沃尔玛等国内外知名企业和现代经营模式，加快中心商贸区、胜利路精品商业带和特色商业街建设，发展连锁业、社区服务业和电子商务，提升鞍山商业现代化水平。二是大力发展文化旅游产业。促进文化产业和旅游产业相结合，加大招商引资力度，重点吸引文化旅游产业项目向千山文化旅游产业带集聚。推进岫岩满族风情园等文化旅游项目建设，大力培育文化产业龙头企业。三是积极发展咨询、评估等各类中介组织，培育壮大行业协会。

在继续支持鞍钢、中冶东建公司等国有企业做大做强的同时，促进民营和外资经济快速发展。一是继续深度推进国有企业和鞍钢附企等集体企业的改革与发展。全力支持鞍钢、中冶东建公司等国有企业大发展、快发展。按照“发展、就业、改革、保障”的思路，积极稳妥地推进鞍钢附企改革与发展。继续按照“四条标准、三个途径、五个专项、两大办法”，争取彻底解决国企历史遗留问题。引导企业股权和资产开放，支持改制企业实施股份制改造和实现倍增发展目标。切实加强国有产权、资产价值、债权、资产保全和企业绩效的监督管理。二是大力发展民营经济。大力支持本地民营企业发展。加强银企交流合作。建立完善民营企业信用制度、融资平台和中介服务体系。积极引导和帮助企业上市。积极吸引域外民营企业来鞍投资发展。全市民营经济主要指标均增长20%以上。三是大力发展外资经济。广泛吸引国际知名大公司、大企业来鞍发展兴业。鼓励中橡公司等现有外资企业增资扩股，吸引更多的外资企业投资鞍山，进一步扩大鞍山外资经济份额。

在继续加快发展的同时，注重环境保护、节能降耗、资源节约和自主创新。一是加大环境保护力度。推进鞍钢一发电等14个大气治理工程。逐步取缔10吨以下燃煤锅炉。实施工业炉窑专项整治。推进鞍钢矿业公司尾矿库抑尘工程。完成矿山复垦50万平方米。保持建成区空气环境质量全年80%以上天数达到国家二级标准。实施水污染和噪声污染治理工程。取缔南沙河、杨柳河沿岸非法选矿，整治滥挖河沙行为。二是强化节能降耗。依法淘汰高耗能的落后工艺和设备。突出抓好重点耗能行业和企业的节能降耗。推广采用新型节能墙体材料和节能设施。强化对机动车、锅炉、电动机等重点耗能设备的定期节能监测。三是推进资源节约。调整好矿产开发与深加工的关系，推进镁砂、滑石等矿产品向精深加工方向发展。整顿矿产资源开发秩序，坚决制止滥采乱挖和资源浪费行为。贯彻实施《鞍山市节约用水条例》。四是进一步提高科技创新能力。调整好引进与消化、吸收再创新的关系。以科研院所为依托、高新技术企业为主体，加快高新技术产业发展。强化企业科技创新主体作用，重点支持企业与高校联建研发中心、中试和产业化基地。落实人才强市战略，积极引进国内外各类人才。

（二）以发展县域经济为重点，大力推进新农村建设

以发展县域经济为重点，以农民增收为目标，以加大投入为保证，推进新农村建设迈出更大步伐。力争县域生产总值和财政一般预算收入分别增长20%以上。

推进工业进园区，发展县域工业。积极争取省财政支持，加大市财政支持力度，扶持各县（市）区加快园区建设，推动工业园区化、产业集聚化。支持海城发展矿产品深加工、钢铁深加工和轻纺工业；支持台安发展设施农业、特色农业及深加工；支持岫岩发展矿产资源开发和深加工、特色农产品和林产品加工业；支持千山区发展钢铁及深加工与产业配套集群。

按照集约化方向，发展县域农业。力争建设一批设施农业村、设施农业镇、设施农业带。全面推进鞍羊路设施农业示范带和海岫路特色农业示范带建设。扩大“一村一品”、“多村一品”规模，大力发展无公害、绿色和有机农产品生产。发展壮大农业产业化龙头企业，大力发展农产品精深加工项目，推进现代农业园区和畜牧养殖小区建设。

推进农村市场建设，发展县域服务业。完善便民服务网络，加强西柳服装城等大型专业市场和物流基地建设。鼓励有条件的村镇发展农事旅游。大力发展农村专业合作组织和农民经纪人队伍。

加大公益性投入，发展县域社会事业。深化农村综合改革。加强农田水利基础设施建设，深化农村小型水利设施使用权流转制度改革。有序推进农村城镇化和村屯改造。新建农村公路137公里，新建4个乡镇客运站。新建农村饮水工程40处，新增自来水受益人口7万人。新增新能源用户4万户。强化农村生态环境保护，高标准绿化村屯100个，实施人工造林5万亩，全面实施鞍辽路等公路绿化工程，做好湿地恢复和保护工作，对东部山区生态加强保护，加强台安西部沙漠化治理。推进花卉苗木等林业产业项目建设。深化集体林产权制度改革。完善农村公益服务体系，确保每个乡镇建一个特色农业技术推广站。新型农村合作医疗参合率达到95%以上。探索建立失地农民养老保险制度，将失地农民纳入社会救助体系。调整和优化农村教育资源配置，推行集中办学模式。继续做好农村“扶困助学”及义务教育阶段“两免一补”工作。

（三）以园区建设为载体，推进大项目、大企业、大产业、大集群建设

坚持以项目为核心，开展项目攻坚年活动，加速构建鞍山经济隆起带。

加大园区建设力度。一是推进园区规模化。以腾达大道为纽带，推进灵山工业区、达道湾工业区、四达物流园区、宁远工业区和汤岗子农高区建设，形成鞍山的“五点一线”,并继续向南延伸，逐步构建鞍海经济带。规划建设千山文化旅游产业带，启动千山水库和南沙河水系建设，推进南沙河沿岸开发。继续拓展高新区发展空间，广泛吸引高新技术产业项目入区发展。二是明确园区发展定位。将高新区建设成为高新技术产业的先导区。将灵山、达道湾、宁远工业区建设成为装备制造、钢铁深加工、化工和新型建材等重化产业的集聚区和提升区。将汤岗子农高区建设成为农副产品深加工和食品、饮料等消费产业的聚集区。将四达物流园区建设成为以钢材、机电五金、汽车零部件等为主的现代物流园区。各县域工业园区要突出资源等比较优势，形成具有特色的产业园区。三是加强园区基础设施建设。以全线贯通“五点一线”道路交通为重点，全面推进园区路网、管网、供电、环保等各种配套设施建设，逐步形成功能齐备、配套完整、设施完善、环境友好的新型工业园区。

以推进大项目、大企业、大产业、大集群建设为重点，全力开展项目攻坚年活动。一是引进大项目。全力引进对鞍山经济社会发展具有牵动作用的战略性重大项目，积极支持鞍钢120万吨1450冷轧板、60万吨煤焦油深加工等6个重大项目建设；推进烨联公司20万吨不锈钢冷轧板、瑞士钢铁公司年产100万吨合金线材、翔鹭化纤公司工业丝和帘子布、腾龙公司热电厂、鞍轮集团120万套全钢丝子午胎等重大项目，全年力争引进亿元以上项目200个。全年滚动推出“四个一批”项目1500个，力争当年开工建设规模以上项目600个。二是扶持大企业。全力支持鞍钢逐步向世界500强企业跨进。打造西洋集团、翔鹭化纤公司、宝得钢铁公司、中冶东建公司4个销售收入超100亿元的企业。打造后英集团、鞍钢重机公司、衡业集团、鞍轮集团4个销售收入超50亿元的企业。打造中橡公司、宁波燎原公司、华冶公司、森远集团等10个销售收入20—30亿元的企业。打造东亚公司、海诺集团等20个销售收入超10亿元的企业。三是发展大产业。按照拉长产业链、进行精深加工发展方向，加快建设全国重要精品钢材基地。按照规模化、集群化、成套化、智能化发展方向，构筑装备制造业基地。按照精深加工发展方向，发展矿产品深加工产业，形成国内最大镁制品深加工产业基地。按照增加品种、提高档次发展方向，加快构建纺织产业基地。四是培育大集群。按照集群效应谋划产业发展格局，以“一个基地、三大产业”为重点，加速建设和形成冶金、装备制造、矿产品深加工、纺织、轻工、化工、建材、医药等八大行业的产业集群。

（四）以解决好人民群众最关心、最直接、最现实的利益问题为着力点，将振兴发展成果惠及广大人民群众

加大政府投入，继续坚持为全市人民办实事，在广泛征求城乡人民群众意见的基础上，再确定若干件实事，让广大群众共享振兴发展成果，共同走上幸福之路。

继续解决好群众安居问题。确保上年动迁的7片25万平方米7400户棚户区居民和28片76万平方米2.1万户危房居民按时回迁。完成剩余的45万平方米1.1万户危旧房居民动迁。加速实施旧楼区改造。

继续解决好群众就业问题。深入开展就业工作规范化管理试点和创建充分就业社区工作。全面建立和完善市区两级劳动力市场。对下岗失业人员、鞍钢附企职工、进城务工和转移就业农民免费提供培训，促进再就业。加大军嫂就业安置力度。继续通过大力发展工业、服务业，创造更多的就业岗位。继续购买公益性岗位，解决困难群体就业问题。继续推进动态实现“零就业家庭”至少1人就业，推进棚户区“零就业家庭”实现“双就业”。着力解决大中专毕业生就业难的问题。全年实现实名制就业10万人以上，城镇登记失业率控制在5.5%以内。

继续完善社会保障体系。扩大社会保险覆盖面，努力实现社会保险应保尽保和保险基金应缴尽缴。继续扩大城市合作医疗保险覆盖面，将鞍钢附企等集体企业职工和不具备参加基本医疗保险的人员逐步纳入城市合作医疗保障范围。做好社会救助工作，规范低保管理，对低保人员实行动态管理下的应保尽保。继续做好城乡贫困家庭的基本生活、子女上学、住房困难等方面的全方位救助。

继续做好供暖、供水、供气、公交等工作。继续加大供暖设施的改造力度，全面解决弃管房无人管理的问题。推进汤河水源二期工程和大伙房供水工程建设，进一步改善城市供水质量。与鞍钢合作建设煤制气项目，努力保证气源供应。增加、完善公交线路和运营车辆。

（五）以发展社会事业为切入点，努力构建和谐鞍山

全面提升社会事业发展水平，努力实现经济社会协调发展。

深入推进创城工作。以创建全国文明城市为统领，巩固国家卫生城市、全国优秀旅游城市、全国双拥模范城市和全国畅通工程一等模范管理城市成果，继续强化国家环保模范城市、国际健康城市和综合治理先进市的创建工作。

优先发展教育事业。推进城乡义务教育均衡发展。完成20所薄弱学校改造任务。全面实施素质教育。依法促进民办教育健康发展。提高普通高中教育质量。扩大农村高中办学规模。大力发展职业技术教育，整合优化全市职业技术教育资源，建设鞍山职业技术教育城。办好辽宁科技大学博士点。改善高等教育办学条件，培养创新型和实用型人才。

着力发展卫生事业。调整优化医疗卫生资源结构，整合全市精神卫生资源和结核病防治资源，改造汤岗子理疗医院，建成中心医院老干部病房。完善社区卫生服务体系。实现29所乡镇中心卫生院用房全部达到卫生部要求标准。加强重大传染病疫情预防和控制。

大力发展文化、体育事业。建立覆盖城乡的公共文化设施网络。做好数字电视整体转换前期准备工作。改造、建设市博物馆、群众艺术馆和图书馆。加大对古老岩石等自然遗产保护力度。发挥现有体育设施作用，加强城乡体育设施建设。广泛开展群众性体育活动，提高竞技体育水平。

进一步完善社会管理。深入开展和谐社区建设，争创全国社区建设示范市。完善应急管理体系，确保高效快捷处置突发事件。完善农产品检测体系和动物卫生监督管理体系，保证食用农产品质量安全。规范各类市场建设，加强市场监管。落实党的民族政策，加大对少数民族地区的扶持力度。做好军转干部和退役士兵安置工作。继续加强消防和安全生产工作。强化社会治安综合治理，加快视频监控电子警务系统建设，严厉打击各类刑事犯罪活动，让人民群众有安全感。

（六）以优化人居环境为主题，提高城市建设与管理水平

加大城市建设和管理力度，促进城乡面貌发生较大变化。

加强城市建设。完成城市总体规划修编，调整土地利用总体规划。推进国电集团两台30万千瓦发电机组的热电厂建设。推进哈大铁路客运专线和岫庄铁路建设。开工建设四达大道等道路，完成腾达大道、曙光路和建材街等道路改造工程，拓宽改造解放路、鞍辽路、红旗路，开工建设东环路、深营路东线隧道，尽快形成鞍山一、二环线。加速居民小区和街巷路改造建设。继续改造玉佛山风景区和二一九、孟泰、永乐公园，增加休闲健身设施。建成眼镜湖和沙河文化公园。完成植树6万株，新增城市绿地75万平方米。搞好山体、桥涵和主要建筑物的亮化。新建、改扩建20处农贸市场，补充完善停车场等公共设施。

加强城市管理。加大市容环境综合整治力度，继续清理广告牌匾和违章建筑，清除城市小广告，粉饰临街建筑和主要建筑，清理占道经营、乱停乱放和乱搭乱挂。严厉打击各类黑公交车、黑出租车。全面规范城区机动车修理、铝塑铁艺加工等业态。规范早晚市管理，推进马路市场退路进厅。

（七）以提高执行力和公信力为重点，加强政府自身建设

进一步转变政府职能，加快推进制度创新、管理创新和机制创新，全面加强政府自身建设。

建设民主法治政府。认真实施《监督法》，自觉接受人大法律监督、工作监督，积极主动向人大及其常委会报告工作。自觉接受政协民主监督，加强与人民政协民主协商。认真办理人大代表议案、建议和政协提案。强化科学决策、民主决策，提高群众参与度。深入实施《全面推进依法行政实施纲要》，严格按照法定权限和程序履行职责。

建设效能责任政府。强化统筹、协调能力，完善公共服务体系建设，加快推行电子政务，提高各级政府的行政效能和决策执行力。强化效能监察，着力解决不作为和乱作为问题，继续加强政府制度建设，做到工作目标明确、权力义务清晰、责任追究到位。

建设为民服务政府。牢固树立执政为民宗旨，畅通政务公开渠道，切实保证人民群众的知情权、参与权和监督权。畅通群众诉求渠道，以解决好实际问题为基本点，以情、以理、依法做好信访工作。深入贯彻实施《行政许可法》，进一步简化行政审批程序，减少审批事项，强化对行政审批的监督管理，规范行政事业性收费和行政处罚自由裁量权，营造良好的投资发展环境。进一步转变工作作风，充分尊重民意、体察民情、尽职尽责，不断造福广大人民群众。

建设廉洁勤俭政府。建立科学严密的权力制约机制，强化“五大中心”建设，确保公开、公平、公正，防止权力寻租。强化审计监督和行政监察。加强公务员队伍建设，弘扬求真务实、廉洁奉公、勤俭节约、艰苦奋斗优良作风，求真务实而不图形式，埋头苦干而不事张扬，力求节俭而不搞铺张，服务为民而不谋私利，切实把经济社会发展和政府各项工作落到实处。

各位代表，回首过去，我们豪情满怀；展望未来，我们信心百倍。我们坚信，经过全市人民的共同努力，鞍山一定会呈现出一派经济繁荣、生活富庶、社会文明、环境优美的新气象。让我们在市委的领导下，在市人大、市政协的监督支持下，锐意进取，埋头苦干，奋发有为，开拓创新，为加速推进我市经济社会又好又快发展而不懈奋斗！
